# Impact of Native Probiotics on Autophagy and Oxidative Stress in Nickel‐Exposed Mice: Insights Into the Gut–Brain Axis

**DOI:** 10.1002/brb3.70399

**Published:** 2025-02-28

**Authors:** Asal Hafezi, Shokufeh Beglari, Shadi Aghamohammad, Mahdi Rohani

**Affiliations:** ^1^ Department of Bacteriology Pasteur Institute of Iran Tehran Iran; ^2^ Department of Biology, Science and Research Branch Islamic Azad University Tehran Iran

**Keywords:** autophagy, gut–brain axis, nickel, oxidative stress, probiotics

## Abstract

**Background:**

The gut–brain axis plays a crucial role in mitigating the adverse effects of environmental agents such as nickel exposure. Nickel, recognized as a heavy metal, poses significant concerns for public health because of its impact on neurological disorders and oxidative stress; consequently, it is prioritized for evaluations of its effects on biological pathways. This study investigates the potential of native probiotic strains to modulate inflammatory and autophagy signaling pathways, which are vital for combating oxidative stress.

**Methods:**

Twenty male NMRI mice were divided into 4 groups randomly and were gavaged with NiCl_2_, followed by administration of a probiotic cocktail that consisted of 4 native probiotic *Lactobacillus* spp. and *Bifidobacterium* spp. Brain tissues from these treated mice were collected to analyze the expression of autophagy‐related genes involved in phagophore, autophagosome, and autolysosome formation using quantitative real‐time polymerase chain reaction (qPCR).

**Results:**

Our findings demonstrated that treatment with this cocktail of native probiotic *Lactobacillus* spp. and *Bifidobacterium* spp. significantly increased the expression of autophagy genes compared to the control group exposed to NiCl_2_ alone. Specifically, there was a notable upregulation in genes associated with autophagic processes, indicating that these probiotic strains effectively activated autophagy pathways in response to nickel‐induced oxidative stress.

**Conclusion:**

The beneficial effects of our native probiotic strains were confirmed through enhanced expression of autophagy genes and reduced neuroinflammation, suggesting their potential as therapeutic agents in mitigating the adverse impacts of nickel exposure on brain health via modulation of the gut–brain axis.

## Graphical abstract

The gut–brain axis plays a crucial role in mitigating the harmful effects of nickel exposure. Nickel, a heavy metal, induces oxidative stress, making it essential to utilize beneficial agents like probiotics. This study examines how native probiotics can influence autophagy and reduce oxidative stress caused by nickel via the gut–brain axis. Our findings indicate that these probiotics enhance autophagy gene expression, providing a potential therapeutic strategy against nickel‐induced brain damage.

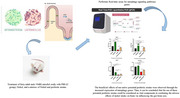

## 1 Background

Nickel (Ni), being classified as a heavy metal, is included among the aforementioned substances, and its intake of more than 0.02 mg/L (in water) could influence one's state of well‐being (Gürel 2017). These heavy metals may pose significant concerns for public health since they are commonly found environmental pollutants, mainly as a result of industrial processes and have been associated with various health issues, including neurotoxicity and oxidative stress (OS) (Anyachor et al. 2022).

The detrimental consequences that arise from the intake of nickel are closely associated with the generation of reactive oxygen species (ROS). When the intestine is infiltrated by nickel, it has the potential to escalate the production of ROS, consequently exacerbating OS (Genchi et al. 2020). OS, in turn, has the ability to induce significant harm to proteins, DNA, and lipids, ultimately leading to the demise of cells (Feng and Wang 2020).

Besides the aforementioned disadvantageous effects, nickel exposure can disrupt the gut microbiota, leading to dysbiosis, which has been linked to alterations in the gut–brain axis, since in recent years, numerous studies have unveiled significant correlations between dysbiosis and impacted organs located physically distant from the gastrointestinal tract, such as the central nervous system (CNS) (Zhu et al. 2024; Rutsch et al. 2020). Also, the dysbiosis can result in increased intestinal permeability, allowing pro‐inflammatory cytokines and neurotoxins to enter circulation, which may affect neuroinflammation and contribute to neurological disorders (Kearns). Additionally, nickel can influence neurotransmitter systems, such as serotonin production in the gut, which is crucial for mood regulation and cognitive functions, thereby potentially impacting behavior and mental health (Martínez‐Martínez et al. 2020). This makes it a relevant metal for studying its effects on biological systems, consequently, it is prioritized for evaluations of its effects on biological pathways that can reveal important insights into the mechanisms underlying diseases associated with dysbiosis and inflammation, potentially leading to novel therapeutic strategies.

As said above, the ingestion of nickel may result in OS; thus, any method that can be employed to counteract OS and mitigate the detrimental effects of nickel consumption is crucial for preserving health. Autophagy is a vital mechanism that plays a crucial role in ensuring cell survival when cells are subjected to various external stresses, such as OS. Autophagy is an essential physiological process in intact cells and a cellular protective mechanism, serving as an intracellular self‐repair mechanism (Qiao et al. 2022). A significant amount of research has demonstrated the connection between dysregulated autophagy and various diseases, such as neurodegenerative disorders and cancers. Within this context, certain metals or combinations of metals have been identified as environmental triggers that can disrupt the regular autophagic process, leading to negative health consequences. Furthermore, investigations have indicated that particular autophagy inhibitors or enhancers have the potential to alter the abnormal autophagic flow induced by prolonged exposure to metals (Li et al. 2023). Recent research has demonstrated that heavy metals not only contribute to OS but also hinder the process of autophagy flux, consequently exacerbating cellular damage. Therefore, the exploration of natural compounds capable of regulating and reinstating autophagy flux represents a promising novel therapeutic avenue (Avila‐Rojas et al. 2019). Consequently, agents that positively affect the function of this pathway could be crucial, especially when an individual faces heavy metal toxicity, such as nickel toxicity.

Probiotics, as living microorganisms, exhibit a remarkable and protective effect on cells and tissue (Rehan et al. 2022). Among the different pathways suggested for the mechanisms of probiotics, the impact on autophagy is an interesting one (Wu et al. 2013). Numerous studies have shown that the administration of probiotics has the capacity to trigger, regulate, or adjust autophagy via the activation of diverse signaling ways (Nemati et al. 2021). Some specific strains of probiotics, for example, *Lactobacillus* spp., have the ability to regulate the balance between apoptosis and autophagy, resulting in enhanced survival of the follicles (Zhu et al. 2017). *Bifidobacterium* is an additional probiotic that enhances the expression of autophagy proteins and fosters their endurance during stress (Nemati et al. 2021). Probiotics exert beneficial effects on signaling cascades, particularly autophagy. Their influence on environmental toxins, such as heavy metals that can cause OS and other adverse effects, has recently attracted considerable attention. While diverse approaches have been used to mitigate the risks posed by these substances, cost and side effects remain significant challenges. Therefore, probiotics may represent a safe and effective strategy for alleviating the harmful effects of heavy metals (Abdel‐Megeed 2021; Al‐Enazi et al. 2020).

Given the potential of probiotics to mitigate the negative effects of heavy metals and their known antioxidant properties, this study aimed to understand how native probiotic strains influence autophagy. Specifically, we investigated whether these probiotics could alleviate nickel‐induced OS in the brain (via the gut–brain axis) by modulating the expression of key autophagy‐related genes.

## 2 Materials and Methods

### 2.1 Bacterial Strain and Growth Conditions and Cocktail Preparation

Four strains of native probiotic strains screened from our previous study were used in the present study named *Lactobacillus brevis* 205, *Lactobacillus mocusae* 226, *Lactobacillus casei* 375, and *Bifidobacterium infantis* 1001. These strains were isolated from healthy people's guts (belonged to the Pasteur Institute of Iran), which were cultured on Man, Rogosa, and Sharpe (MRS) broth (Merck, Germany) and incubated overnight at 37°C. The next day, bacterial cells were washed three times using PBS buffer and centrifuged (6000 rpm, 5 min, 4°C). Pellets were suspended in an adequate amount of PBS. The bacterial concentration of each strain was adjusted to 7.5 × 10^8^ cfu/mL to make the probiotic cocktail. The bacterial stock of all strains was prepared in glycerol‐ MRS broth and kept at –20°C for further examinations (Beglari et al. 2022).

### 2.2 Nickel Chloride Solutions

A stock solution of 397 g/L (1.67 M) nickel chloride was created by dissolving 50 g of hexahydrate nickel chloride (NiCl_2_.6H2O) in 100 mL of distilled water. Subsequently, a solution with a concentration of 9.6 g/L (0.04 M) was derived from the stock solution.

### 2.3 Animal Model

Male NMRI mice (*n* = 20) with an average weight of 25–30 g were purchased from the production unit of Pasteur Institute of Iran (Karaj, Iran). The male mice were chosen in order to minimize confounding factors, including hormonal fluctuations that could occur in female mice. The mice were grouped into four groups, according to G*Power (Faul et al. 2009). Mice were kept in stainless steel cages, having 10 mice each, and were fed with mice food and water ad libitum. Our groups consisted of the following: control group (C), nickel group (N), probiotic cocktail group (P), and nickel/probiotic cocktail group (NP).

### 2.4 Experimental Groups

The control group received distilled water (DDW). In the N group, each mouse was given 40 µL of 0.04 M of NiCl_2_ daily. The mice in the P group were fed with 100 µL of probiotic cocktail containing *Lactobacillus* and *Bifidobacterium* spp. (the prepared probiotic cocktail had a concentration of 7.5 × 10^8^ cfu/mL of each bacterial strain, with an equal volume of each in PBS buffer as a carrier), and the mice in the NP group were fed with 100 µL of probiotic cocktail one hour after taking 40 µL of 0.04 M NiCl_2_. This timing was chosen to allow for immediate intervention following the OS induced by nickel, aiming to maximize the protective effects of the probiotics. The mice were administered nickel chloride and the probiotic cocktail orally for a period of 60 days (Lamtai et al. 2020; Beglari et al. 2023) via a gavage using a smooth plastic needle. All procedures were approved by the Ethics Committee of the science and research branch of the Islamic Azad University of Tehran, Iran (IR.IAU.SRB.REC.1399.106). All methods were carried out in accordance with ARRIVE guidelines.

### 2.5 Mice Scarification and the Tissue Collection

**FIGURE 1:**
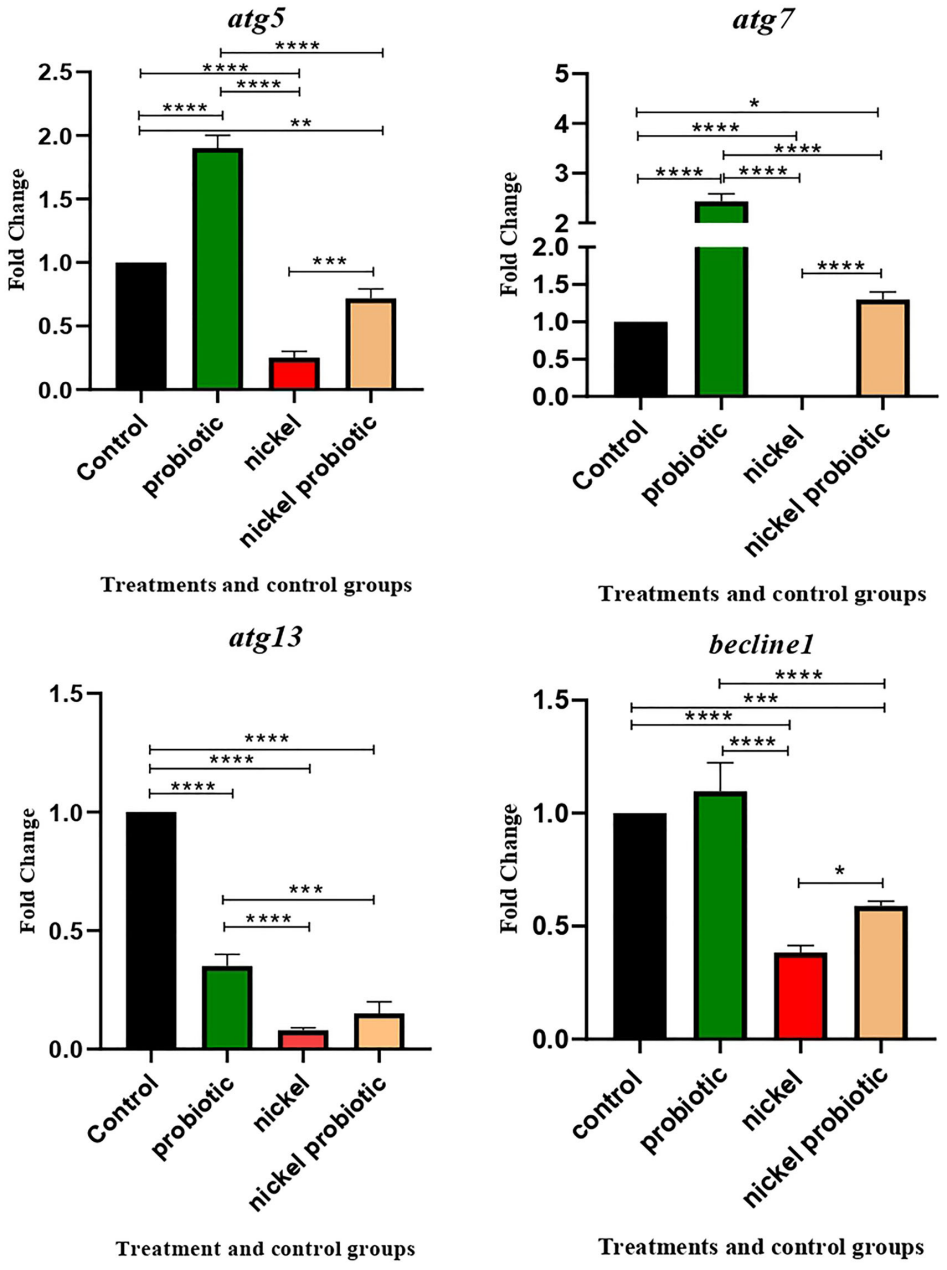
Relative gene expression (mean fold change) of (A) *atg13*, (B) *atg5*, (C) *atg7*, and (D) *beclin1* in the different groups of treatments. Data were normalized with *gapdh*. Data were represented as mean SD. Data were considered as statistically significant when *p* < 0.05 (*p < 0.05, **p < 0.01, ***p < 0.001, and ****p < 0.0001). It should be noted that *p* values were corrected for multiple comparison using Tukey's post hoc test.

After the final examination, all mice were sacrificed by cervical dislocation, following which the brain tissue was expeditiously extracted utilizing a board cooled with ice. This was followed by a thorough cleansing with phosphate buffer, and the samples were subsequently preserved at a temperature of −80°C until they were employed. It is important to highlight that the overarching pattern of methodology is illustrated in Figure 1.

### 2.6 RT‑PCR of Autophagy Related Genes

According to the manufacturer's instructions, the total RNA was extracted using a total RNA extraction kit (Favorgen Biotech Corp, Taiwan). The quantity and quality of the purified RNA were determined using a NanoDrop1000 UV–Vis Spectrophotometer by measuring absorbance at 260/280 nm. According to the manufacturer's instructions, 10 ng of the RNA samples was used, and the cDNA template was synthesized with the cDNA synthesis kit (Yekta Tajhiz Azma Co, Iran). The online Primer‐Bank website (http://pga.mgh.harvard.edu/prime.rbank) was used to choose the qPCR primers (Table 1).

**TABLE 1:** Primer sequences used in this study.

Gene	Primer sequence [5′ > 3′]	Primer bank ID
*atg5* F	AGCCAGGTGATGATTCACGG	26360254a1
*atg5* R	GGCTGGGGGACAATGCTAA	
*atg7* F	TCTGGGAAGCCATAAAGTCAGG	358679368c1
*atg7* R	GCGAAGGTCAGGAGCAGAA	
*atg13* F	TTCCCCGACGGGTTCAGAT	357527428c1
*atg13* R	GGCCTTCTTTGCTTCATGGG	
*beclin1* F	TCAGCCGGAGACTCAAGGT	142352751c3
*beclin1* R	CACAGCGGGTGATCCACATC	
*gapdh* F	AGGTCGGTGTGAACGGATTTG	6679937a1
*gapdh* R	TGTAGACCATGTAGTTGAGGTCA	

The primers were tested using gradient PCR to get an appropriate annealing temperature. For qRT‐PCR 2 µL of cDNA was added to SYBR Green master mix (RealQ Plus Master Mix Green, Amplicon A/S, Denmark) and made up to a final volume of 20 µL with RNase free water. The mRNA of the studied genes was quantified with the ABI Step One Plus detection system (Applied Biosystems, USA) using the SYBR Green master mix (Amplicon Bio, Denmark). All the reactions were performed in duplicate. The formula RQ = 2^−ΔΔCt^ was used to get relative gene expression in the comparative CT method (Aghamohammad et al. 2022). An appropriate internal control gene, glyceraldehyde 3‐phosphate dehydrogenase (*gapdh*), was selected as a housekeeping gene to normalize the data.

### 2.7 Statistical Analysis

Graphical and statistical analyses were performed using GraphPad Prism 8.0 (GraphPad Software Inc., CA, USA) software to compare different groups. Statistical differences between multiple groups, including the control group (C), nickel group (N), probiotic cocktail group (P), and nickel/probiotic cocktail group (NP), were determined using ordinary one‐way ANOVA followed by Tukey's post hoc test for multiple comparisons. Results were expressed as mean ± standard deviation (SD), with statistical significance set at *p* < 0.05.

## 3 Results

Two comparisons are presented to show the impact of native potential probiotic strains on gene regulation. The first comparison contrasts the control group (as a normal situation) with other treatments. The second comparison, N and NP groups, aimed to determine whether the probiotic strains offered protection against nickel‐induced autophagy dysregulation.

**FIGURE 2:**
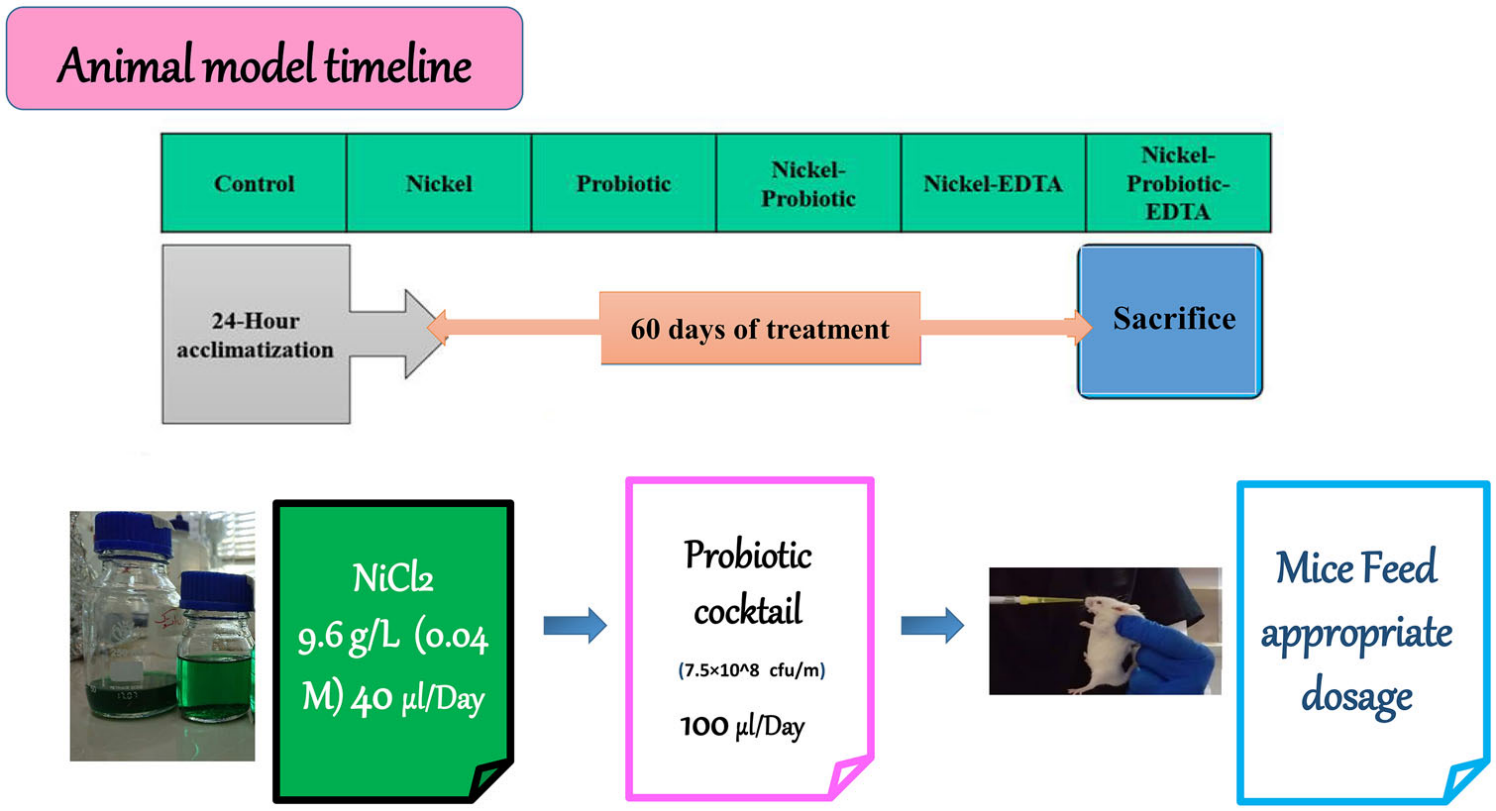
The overarching pattern of methodology.

Autophagy‐related gene expression data is shown in Figure 2. A comparative analysis of *atg5* gene expression between various groups and the control group showed notable findings. The comparison between the N group and control showed that nickel could significantly decrease gene expression (*p* < 0.0001). In the P group, the analysis demonstrated that using our native potential probiotic strains (*Lactobacillus* and *Bifidobacterium* spp. strains) could significantly increase the expression level of *atg5* (*p* < 0.0001). However, upon analyzing the NP group, it was observed that the consumption of our native potential probiotic strains one hour following the administration of nickel resulted in a reduction in the gene expression level (*p* < 0.01). Also, the comparison of the N group and NP group revealed that our native potential probiotic cocktail could significantly increase the expression of the *atg5* gene in comparison to the N group (*p* < 0.001).

In the *atg7* study, the findings from comparing gene expression between different groups and the control group indicated a significant decrease in gene expression levels due to nickel treatment (N group), reaching a level of zero (*p* < 0.0001). Additionally, our native potential probiotic cocktail (P group) demonstrated a significant increase in the expression level of the *atg7* gene (*p* < 0.0001). Moreover, when our native potential probiotic strains were administered one hour after nickel treatment (NP group), there was a slight increase in the expression level of *atg7* (*p* < 0.05). Furthermore, the comparative analysis of the NP and N groups revealed that our *Lactobacillus* and *Bifidobacterium* spp. were able to significantly enhance the gene expression level (*p* < 0.0001).

A comparative analysis of gene expression of the *atg13* gene was conducted between different groups and the control group. The results revealed that in the N group, the gene expression was significantly decreased by the presence of nickel (*p* < 0.0001). Similarly, in the P group, the utilization of our native probiotic strains led to a decrease in the expression level of *atg13* (*p* < 0.0001). In the NP group, where probiotic strains were administered one hour after nickel exposure, gene expression was significantly reduced (*p* < 0.0001) compared to the control group. However, there was no significant difference in gene expression between the N and NP groups.

In relation to the expression of the *beclin1* gene, nickel significantly decreased *beclin1* expression in the N group compared to the control group (*p* < 0.0001). There were no significant changes in gene expression between the P and C groups. However, in the NP group, the expression level experienced a significant decrease (*p* < 0.001). In addition, it is worth mentioning that the utilization of our native potential probiotic cocktail one hour after administering nickel treatment in the NP group resulted in an increase in the mRNA level of the gene expression (*p* < 0.05) when compared to the N group.

## 4 Discussion

The impact of gut microbiota on the brain, referred to as the microbiota–gut‐brain axis, primarily consists of the nervous, endocrine, and immune pathways (Liang et al. 2018). The gastrointestinal tract is exposed to food and environmental antigens, along with the indigenous gut microbiota and the molecular by‐products it generates (Agirman et al. 2021). Nickel, being a transition element, is extensively distributed in the environment, and ingesting it in quantities exceeding acceptable levels can significantly impact one's well‐being (Genchi et al. 2020). One of the negative consequences of consuming nickel is the stimulation of ROS (Guo et al. 2019), and this phenomenon could become more challengeable due to the imbalance in the gut that can also affect the brain's OS (Dumitrescu et al. 2018). Consequently, brain cells can regulate the impact of OS by activating the autophagy mechanism (Qiao et al. 2022).

This study demonstrated that native probiotic strains significantly influence autophagy, potentially mitigating the negative effects of nickel intake. The *atg13*, *atg5*, *atg7*, and *beclin1* genes, key components of the autophagy signaling pathway, play distinct roles in various stages of this process (Mizushima 2018). *atg13*, together with other components such as *ULK1*, plays a crucial role in the initiation phase. *beclin1* could play a significant role in the nucleation phase, while *atg12* and *atg7* are essential components in the elongation phase (Li et al. 2020). Therefore, in this current study, we managed to thoroughly analyze nearly the entire pathway by investigating the different stages of autophagy. The most recent findings of this evaluation revealed significant findings. Based on these findings, it was observed that our native probiotic strains were capable of enhancing the expression level of autophagy genes in comparison to the control group, with the exception of *atg13*. Highlighting the role of these genes, including *beclin1*, for example, our results indicate that probiotic strains enhance *beclin1* expression, which supports the notion that probiotics may stimulate autophagy through this pathway. This finding aligns with literature (Wu et al. 2019; Nemati et al. 2021) suggesting that probiotics can activate autophagy via *beclin1* upregulation, leading to improved cellular resilience against stressors like nickel. Emphasize that ATG13 is a crucial component in the initiation phase of autophagy, functioning alongside ULK1 to trigger the autophagic process. Despite our findings showing an increase in other autophagy‐related genes, the lack of significant change in *atg13* expression suggests a potential regulatory mechanism or compensatory pathway that warrants further investigation. This could indicate that while other genes are upregulated, *atg13* may be modulated differently under nickel exposure or probiotic treatment conditions. Overall, this information suggests that the utilization of probiotic strains may have advantageous effects even in the absence of harmful agents, such as nickel. On the other hand, our evaluation showed that the nickel intake significantly lowered autophagy gene expression levels, specifically in the expression level of *atg7*, which become zero after consuming nickel. Nevertheless, when we simultaneously utilized our potential native probiotic strains with nickel, the expression of these genes has significantly increased.

The combination of this data, along with our recent phenotypic investigation that is not the aim of the current study and therefore the data are not shown here, considering the effects of nickel intake, demonstrated significant findings. The recent investigations revealed that ingesting nickel caused an increase in OS enzymes, specifically catalase. Additionally, abnormal behavior was observed. In contrast, probiotic strains led to reduced levels of OS enzymes and improved behavioral outcomes (data not shown), findings supported by previous research. Ijomone et al. reported that cognitive and motor functions exhibited impairment subsequent to nickel interventions. Furthermore, alterations in the levels of OS markers and nitric oxide were observed as a consequence of nickel treatments (Ijomone et al. 2018). On the other hand, as per the research conducted by Goyal and colleagues, the utilization of probiotic strains such as *Bacillus*, *Lactobacillus*, and *Bifidobacterium* may engage diverse mechanisms in order to counteract the toxic effects of nickel in mice (Goyal et al. 2019). To further understand the observed effects of native probiotic strains, we focused on the autophagy pathway, hypothesizing that it plays a key role in mitigating nickel‐induced toxicity. By doing so, our aim is to shed light on the intricate molecular mechanisms that underlie the positive effects of our native potential probiotic strains in mitigating the harmful consequences that are associated with the intake of nickel, particularly OS. Considering this particular point in the ongoing study, we have administered both nickel and our native strains of probiotics through gavage, which involves force‐feeding the substances directly into the gut, and subsequently, we have obtained brain tissue samples for the purpose of pursuing and advancing the molecular investigation on said tissue. It becomes evident that this aspect is noteworthy due to the fact that we have successfully demonstrated and illustrated the significant role that the gut–brain axis plays in various physiological and biochemical processes.

The current investigation possesses certain limitations. For instance, in the case of *atg13*, in comparison to the control group, probiotic treatment resulted in a decrease in mRNA levels of gene expression. This implies that further examination of the appropriate dosage of probiotics is required to ascertain its efficacy in the body. Conversely, when utilizing our native potential probiotic strains, no substantial difference was observed in the expression levels of *atg13* between the N and NP groups. This phenomenon may be attributed to various factors that require further investigation. Also, it would be worthwhile to explore other genes of autophagy and a greater quantity of mice during future research endeavors. Furthermore, in our current investigation, we acknowledge that the variability in individual probiotic strains can significantly impact their efficacy and the overall outcomes of the study. Different strains may exhibit distinct mechanisms of action and varying levels of effectiveness in modulating autophagy and OS. In addition, the long‐term effects of probiotic supplementation are indeed an important consideration, as prolonged exposure may yield different results compared to short‐term administration.

## 5 Conclusion

In summary, our investigation has demonstrated an increase in autophagy in brain cells following our native potential probiotic treatment. Generally, the assessment of probiotics' specific molecular impacts on autophagy provides a clear understanding of how probiotics regulate and reduce OS. Utilizing these effective potential strains of probiotics could be considered a vital strategy for alleviating the detrimental effects of exposure to environmentally harmful agents, such as nickel, and combating OS to minimize the anticipated side effects in the whole body via a gut–organ axis, such as the gut–brain axis.

## Author Contributions

**Asal Hafezi**: writing – original draft, methodology. **Shokufeh Beglari**: methodology. **Shadi Aghamohammad**: writing – review and editing, visualization, validation, formal analysis. **Mahdi Rohani**: writing – review and editing, supervision, data curation.

## Ethics Statement

This study was carried out in accordance with the Declaration of Helsinki, and it was approved by the Ethics Committee of the science and research branch of the Islamic Azad University of Tehran, Iran (IR.IAU.SRB.REC.1399.106).

## Conflicts of Interest

The authors declare no conflicts of interest.

### Peer Review

The peer review history for this article is available at https://publons.com/publon/10.1002/brb3.70399

## Data Availability

The datasets generated during and/or analyzed during the current study are available from the corresponding author on reasonable request.
